# Influence of music on the hearing and mental health of adolescents and countermeasures

**DOI:** 10.3389/fnins.2023.1236638

**Published:** 2023-08-03

**Authors:** Li Chen

**Affiliations:** Hubei Provincial Committee of the Chinese Communist Youth League, Wuhan, China

**Keywords:** adolescents, mental health, music influence, hearing, review

## Abstract

This review elaborates on the influence of music on the psychological well-being of adolescents, covering the potential advantages, drawbacks, and necessary strategic interventions associated with music exposure. Initially, we characterize music and delve into a multifaceted classification system. Music, as a pervasive art form, is categorized based on regional and national parameters, and it also distinguishes through the assorted genres and traits. The mental well-being of adolescents is significantly influenced by music through mechanisms such as the facilitation of emotional expression and regulation, fortification of social bonds and the sense of belonging, as well as the fostering of creativity and cognitive development. Nonetheless, music, if misused or associated with inappropriate content, could elicit a spectrum of issues ranging from auditory impairment, diversion of attention, addiction tendencies, to the induction of negative emotions. To counteract these potential hazards, we propose several mitigation strategies including the selection of appropriate music styles, the establishment of a wholesome music environment, the promotion of the constructive role of music education, and fostering active participation in music activities among the youth. In conclusion, we underscore the necessity of a collaborative endeavor from all sectors of society to ensure a healthy music environment for the youth, which in turn would enhance the positive influence of music on the mental health development of this population.

## 1. Introduction

Music, as an immersive and broad artistic medium, plays a pivotal role in the lives of adolescents. This stage of life, commonly known as adolescence, is viewed as a crucial period for psychological, social, and cognitive development; the role of music within this framework is significant. As per the research conducted by Ouergui et al. (2023), youth display a high frequency of interaction and deep engagement with music. In the contemporary digital age, music is ubiquitous. Whether via streaming services, social media platforms, or traditional radio and television, music is woven into the fabric of young people’s lives. This pervasive presence of music provides adolescents with a means to express themselves and articulate their emotions; furthermore, it has emerged as a crucial avenue for their social interactions.

Numerous adolescents opt to convey their identities and emotions by curating and sharing music playlists (Taruffi, 2021; Feneberg et al., 2023). This behavior extends beyond the expression of their musical esthetics; it mirrors their quest for resonance and the establishment of social relationships through music. For instance, a melancholic playlist might communicate an adolescent’s low mood, whereas an energetic playlist can depict their elation and positivity. Moreover, sharing these playlists encourages youth to find shared interests among friends and peers, thereby enhancing social connections. Concurrently, music plays a key role in shaping adolescents’ identity and self-perception (McFerran et al., 2010; Haeyen and Noorthoorn, 2021). During this phase, adolescents engage in the process of discovering and molding their identities; the music they select and appreciate often reflects their values, beliefs, and self-concepts. This journey of self-recognition and self-expression through music significantly affects adolescents’ mental health and development.

In recent years, there has been an escalating focus on research exploring the influence of music on adolescents’ mental health. For example, Knoerl R and Neal-Barnett A discovered that youth frequently utilize music as a coping mechanism for their emotions, such as alleviating stress and enhancing mood (Neal-Barnett et al., 2019; Knoerl et al., 2022). Further, several studies have indicated that collaborative musical activities (like choir participation or orchestra) can boost adolescents’ social skills and self-esteem (Porter et al., 2017). However, some research has also highlighted potential risks associated with music, including hearing damage and distraction (Halevi-Katz et al., 2015). These studies collectively underscore the profound influence of music on the mental health of adolescents.

The aim of this article is to review the existing research on the effect of music on the psychological health of adolescents, with a specific emphasis on the benefits and potential hazards of music and strategies for ensuring that adolescents can safely enjoy the psychological benefits of music. This review hopes to offer practical suggestions for parents, educators, and community workers and to guide future research in this domain.

## 2. Definition and classification of music

### 2.1. Definition of music

Music, as a universal form of human expression, has a well-known definition, but it has multiple interpretations in terms of substance and artistic features. In its essence, music is a conscious and organized expression of sound and silence (Tervaniemi, 2023). It involves elements such as melody (a musical phrase formed by a series of notes), rhythm (the organization and duration of notes), harmony (two or more notes sound simultaneously), and color (the characteristics and sensations of music, often contributed by specific instruments or sound sources).

In terms of artistic features, music is regarded as a universal human language that can convey emotions, express ideas, provide comfort, and cross cultural boundaries. It is an art form with high expressiveness and depth, through which we can experience various aspects of life (Mastnak, 2016).

### 2.2. Classification of music

The diversity of music is reflected in its broad classification. Classified by region and country, music from different regions reflects their respective cultural characteristics and traditions. For example, Chinese music is famous for its unique pentatonic scale, strong expressiveness, and rich variety of instruments (Wang et al., 2021; Su and Kong, 2023). The characteristics of Indian music are complex rhythmic structures, such as Tala, and improvisation based on the scale (Raga; Fletcher, 1915; Sharma et al., 2021). Western classical music is known for its rigorous formal structure, rich harmony, and complex note textures (Georges, 2017; Lepping et al., 2019).

Another classification method is based on the genre and characteristics of music. Classical music usually refers to music created between 1650 and 1900, with carefully crafted compositions and rich expressive power. Pop music is the most common type of music since the mid-20th century, characterized by simple melodies, easily accepted lyrics, and strong rhythms (Ren, 2021). Jazz originated in the early 20th century in the United States and is known for its unique rhythm (such as swing rhythm) and extensive improvisation (Roe and Lysaker, 2023). Rock music originated in the United States in the 1950s, and it has had a widespread impact worldwide due to its strong rhythm and gripping guitar solos (Bogt et al., 2021).

Each type of music has its unique characteristics and forms of expression, providing people with various ways of expression and experience. The richness and diversity of music not only reflect human creativity but also provide a powerful tool that can influence and shape our psychological state.

## 3. Benefits of music

### 3.1. Facilitates expression and regulation of emotions

Music has long been recognized as a potent medium for expressing emotions. It enables individuals to convey a wide spectrum of complex emotions. For instance, fast-paced, high-intensity music may signify happiness and excitement, whereas slow-paced, low-key music can indicate sadness and frustration (Dovorany et al., 2023). Through creating and listening to music, adolescents can easily comprehend and manage their emotions. Moreover, music serves as an instrument for emotional regulation, assisting individuals in alleviating stress, mitigating anxiety, and bolstering self-confidence (Aalbers et al., 2017). Research indicates that listening to music can lower cortisol levels (a stress hormone), thereby reducing stress (Emami et al., 2023). The theory of self-efficacy suggests that participation in music activities (such as learning an instrument or singing) can enhance individual self-confidence and self-esteem (Paolantonio et al., 2020).

### 3.2. Enhances social connections and sense of belonging

Music also provides social benefits, as it serves as a shared interest and experience and fosters a sense of connection and belonging. For instance, sharing musical tastes can strengthen friendships among young people and promote the formation of social identity (Yifan Zou and Wang, 2021). Additionally, participation in group music activities (such as orchestra or choir) can help adolescents develop teamwork skills and provide a robust sense of belonging and shared accomplishment (Cores-Bilbao et al., 2019).

### 3.3. Fuels creative and intellectual development

Music is considered a vital source of creativity. Through music composition and improvisation, teenagers can freely explore and express their thoughts, emotions, and imagination (Xia et al., 2023). Moreover, studies have shown that music training can positively affect intellectual development, particularly improvements in musical training correlated with mathematical, logical, and spatial intelligence (Kim and Chung, 2023). Although the specific influence of music on intellectual development continues to be studied, evidence suggests that adolescents involved in music activities perform better academically than those who are not.

## 4. Potential drawbacks of music

Despite the widely researched and acknowledged benefits of music, its potential disadvantages, particularly when improperly used, must be recognized.

### 4.1. Inappropriate usage of music

Excessive and improper use of music may contribute to hearing damage. Long-term exposure to high-volume music, especially through headphones or earbuds, increases the risk of harmful noise exposure, potentially resulting in temporary or permanent hearing damage (Hanson and Fearn, 1975). As a result of their limited self-regulation, adolescents may unconsciously listen to loud music, endangering their hearing health. Consequently, teenagers must be educated on safe music listening habits, including reducing volume, limiting listening duration, and using safe listening devices (Gopal et al., 2019).

Additionally, excessive music listening may distract attention, thereby affecting academic and work performance (Chen et al., 2023). Some argue that music enhances work efficiency, but research suggests that listening to lyrical music can distract attention, particularly when performing tasks requiring language processing (Vasilev et al., 2018). Furthermore, overreliance on music might lead to habitual addiction, which is characterized by an uncontrollable urge to listen to music, potentially affecting daily life negatively.

### 4.2. Potential negative effect of music content

The content of music may induce or exacerbate negative emotions and behaviors. For instance, lyrics containing violence, sex, discrimination, and negativity may negatively influence adolescents (Wang and Jiang, 2022). Studies have demonstrated that young people often interpret and internalize lyrics more than adults do; thus, the youth are more susceptible to these negative influences than adults (Martino et al., 2006).

**Figure 1:**
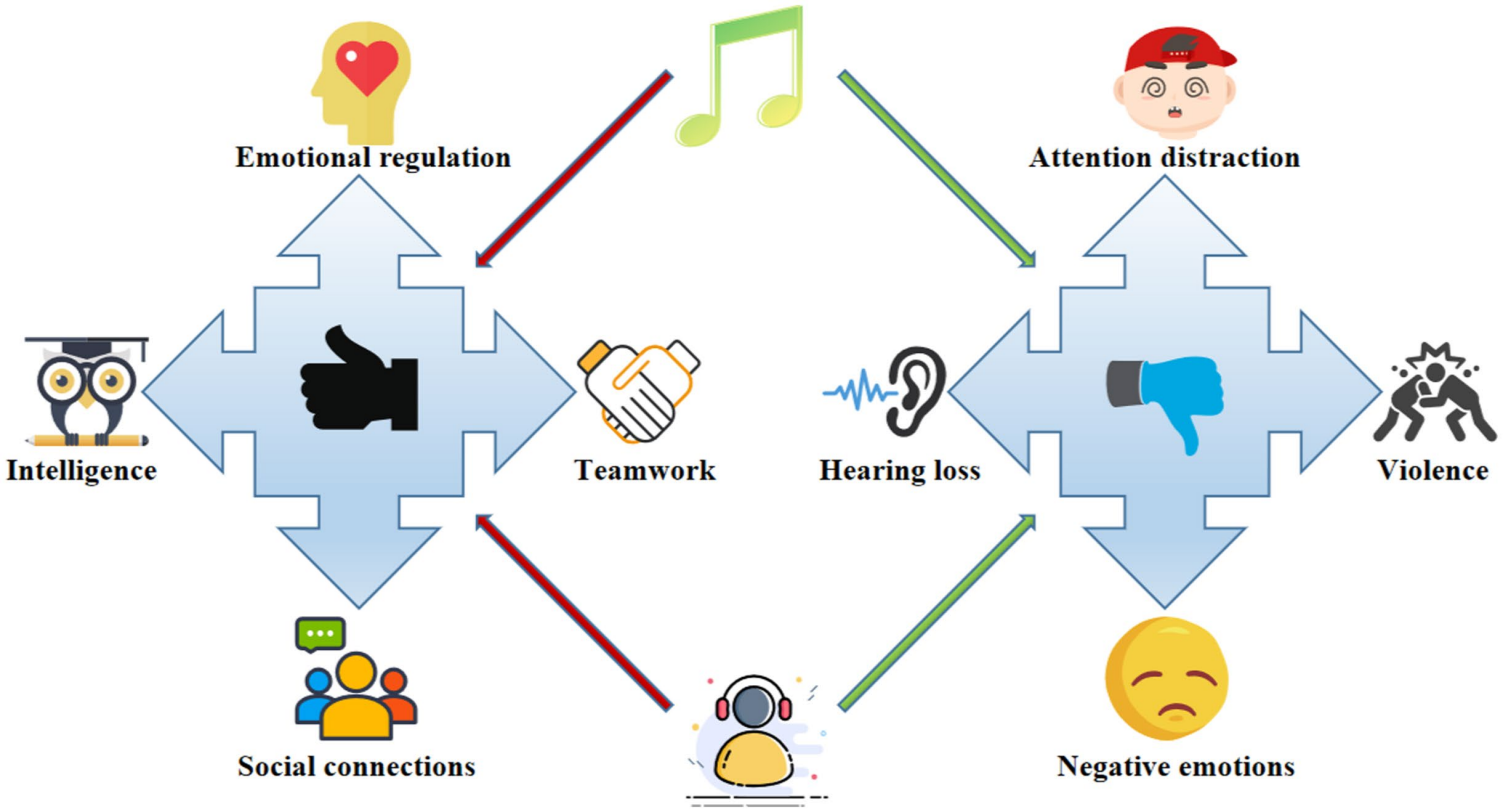
Schematic of music on the mental health of youth.

The emotional tone of music may influence the listeners’ emotional state. Some studies have indicated that listening to melancholic and intense music may induce or exacerbate negative emotions (Hahn et al., 2022). Though young individuals may use such music to regulate or express their emotions, in the long run, excessive exposure to such music may detrimentally affect their mental health (Figure 1).

## 5. Implementing effective coping strategies

### 5.1. Selecting appropriate music genres

For adolescents, the choice of an appropriate music genre is critical, as it can satisfy both their personal preferences and psychological needs. This selection process should prioritize the adolescents’ preferences and psychological needs and consider the esthetic value and educational significance of music. For instance, according to Maslow’s hierarchy of needs theory, music can fulfill physiological needs (such as relaxation and stress relief), safety needs (like fostering a sense of security), social needs (enhancing belongingness and interpersonal relationships), esteem needs (expressing oneself and receiving acknowledgment), and self-actualization needs (such as creation and performance; Healy, 2016).

Furthermore, the esthetic value and educational implications of music should not be overlooked. Esthetic studies illustrate that music can trigger profound emotional experiences and spiritual exchanges and offer beauty appreciation. Education research suggests that music education can enhance holistic adolescent development, including intellectual, emotional, social, and moral dimensions (Ouergui et al., 2023). Thus, we should motivate teenagers to choose music of high esthetic and educational value, including classical music, ethnic music, and world music.

### 5.2. Establishing a healthy music environment

To safeguard adolescents’ auditory health, parents must monitor volume and duration and avoid prolonged exposure to loud music. In addition, the content of music, particularly those expressing negative emotions, violence, discrimination, and other harmful elements, should be regulated. These responsibilities require the collaborative efforts of families, schools, and society at large. The home, being a teenager’s first classroom, should see parents educating adolescents about the appropriate use of music, including volume control, time management, and selection of positive and healthy music content. Schools, as the secondary classroom, should guide adolescents toward appreciating high-quality music, thereby positively impacting music education. The society, being the broader classroom, should offer abundant music resources and services to cultivate a healthy musical environment for adolescents (Kortesoja et al., 2023).

### 5.3. Advocating positive music education

Music education plays a pivotal role in fostering adolescents’ mental health. First, we should prioritize the design and teaching methods of music courses. Music syllabi should encompass diverse facets of music, including music theory, instrumental performance, vocal training, music appreciation, and music creation. Teaching methods should emphasize student-centric approaches and inspire active participation, exploration, and creative thinking. Innovative teaching techniques, such as project-based learning, problem-based learning, and cooperative learning can pique adolescents’ interest and enhance their learning efficacy (Lei, 2022). Second, we should expand the accessibility of music education resources. This expansion includes providing a plethora of resources like music books, music software, and music websites and organizing a variety of music activities such as concerts, music competitions, and music festivals.

### 5.4. Encouraging active participation in music activities

Music activities offer an excellent platform for teenagers to hone their musical skills, showcase their talents, and relish the joy of music. Adolescents should be encouraged to actively participate in various music activities, like learning musical instruments, partaking in performances, and organizing music clubs. These activities not only enhance the music literacy of adolescents but also boost their social, organizational, and leadership skills. Moreover, the innovative spirit in adolescents must be stimulated, and they should be encouraged to engage in music creation, adaptation, and research. These activities allow adolescents to comprehend music profoundly, express music freely, and actively engage in music, thereby enhancing their mental health (Perkins et al., 2016).

## 6. Conclusion

In this systematic review, we explored the intricate relationship between music and the mental health of adolescents. The influence of music on adolescent mental health is significant, encompassing areas such as emotional expression and regulation, fostering social connections and a sense of belonging, and promoting creativity and intellectual growth. Music offers adolescents an avenue to articulate their identity and emotions while assisting in the development of their social networks, creativity, and cognitive abilities. Conversely, the mental health status of adolescents influences their interaction with and perception of music. Adolescents may select different music genres in alignment with their emotional state and psychological requirements. For instance, during periods of stress or unhappiness, they may gravitate toward music that provides comfort or reflects their current emotional state.

This bi-directional relationship underscores the significance of music in the realm of adolescent psychological health, and it contributes fresh perspectives and ideas for future research. Possible future research areas could include studying how adolescents utilize music to manage their psychological stress or investigating ways to enhance their mental health through music education (Wang et al., 2022). Although music confers a multitude of benefits on adolescent mental health, inappropriate utilization of music may have detrimental effects. High-volume music can lead to auditory damage, and excessive music engagement may cause attention dispersion or contribute to addictive tendencies. Moreover, some music content can instigate negative emotional responses in adolescents, encompassing themes of violence and discrimination (Wang and Jiang, 2022). Thus, adolescents should enjoy music in moderation and proactively mitigate possible risks. Parents and teachers bear the responsibility of instructing adolescents on appropriate music consumption, including adjusting suitable volume, managing listening duration, and selecting positive and health-promoting music content. These strategies can help adolescents evade the negative implications of music while maximizing its positive influences, thereby enhancing their mental health status.

The establishment of a healthy music environment is pivotal in promoting adolescent mental health; this goal can be achieved through the collective efforts of families, schools, and society. Homes should provide safe and inviting music environments for adolescents. Schools should be equipped with music education resources and opportunities. Society should facilitate music activities and services to cater to adolescents’ musical needs. Further, adolescents should be encouraged to actively engage in music activities, such as learning instruments and partaking in musical performances. These activities not only foster adolescents’ musical aptitude and knowledge but also hone their social skills, boost self-confidence, and stimulate creativity (Phillips et al., 2019). In this process, the role of music educators is paramount. They bear the responsibility of imparting not only musical knowledge and skills to the adolescents but also helping them understand the spiritual value of music. They should facilitate the understanding of how music affects their emotions and mental states, as well as how to employ music as a tool to enhance their mental health (Steele and Young, 2011).

In conclusion, music significantly influences adolescent mental health. A concerted effort at all levels is required to cultivate a healthy musical environment for adolescents to optimally harness the positive influence of music and avert its potential negative effects.

## Author contributions

The author confirms being the sole contributor of this work and has approved it for publication.

## Conflict of interest

The author declares that the research was conducted in the absence of any commercial or financial relationships that could be construed as a potential conflict of interest.

## Publisher’s note

All claims expressed in this article are solely those of the authors and do not necessarily represent those of their affiliated organizations, or those of the publisher, the editors and the reviewers. Any product that may be evaluated in this article, or claim that may be made by its manufacturer, is not guaranteed or endorsed by the publisher.

## References

[ref1] Aalbers S. Fusar-Poli L. Freeman R. E. Spreen M. Ket J. C. Vink A. C.. (2017). Music therapy for depression. Cochrane Database Syst. Rev. 11:CD004517. doi: 10.1002/14651858.CD004517.pub3, PMID: 29144545 PMC6486188

[ref2] Bogt T. T. Hale W. W. 3rd Becht A. (2021). “wild years”: rock music, problem behaviors and mental well-being in adolescence and Young adulthood. J. Youth Adolesc. 50, 2487–2500. doi: 10.1007/s10964-021-01505-0 34633600 PMC8580930

[ref3] Chen F. Xue H. Wang M. Cai Z. Zhu S. (2023). Hearing care: safe listening method and system for personal listening devices. Int. J. Environ. Res. Public Health 20. doi: 10.3390/ijerph20032161, PMID: 36767527 PMC9915878

[ref4] Cores-Bilbao E. Fernández-Corbacho A. Machancoses F. H. Fonseca-Mora M. C. (2019). A music-mediated language learning experience: Students' awareness of their socio-emotional skills. Front. Psychol. 10:2238. doi: 10.3389/fpsyg.2019.02238, PMID: 31636585 PMC6787270

[ref5] Dovorany N. Brannick S. Johnson N. Ratiu I. LaCroix A. N. (2023). Happy and sad music acutely modulate different types of attention in older adults. Front. Psychol. 14:1029773. doi: 10.3389/fpsyg.2023.1029773, PMID: 36777231 PMC9909555

[ref6] Emami A. Theorell T. Kim H. Berglund L. Hallinder H. Engström G. (2023). Assessing stress using repeated saliva concentration of steroid hormones in dementia care dyads: results from a controlled pilot care music intervention. Ups. J. Med. Sci. 128. doi: 10.48101/ujms.v128.9340 PMC10231046 37265585

[ref7] Feneberg A. C. Stijovic A. Forbes P. Lamm C. Piperno G. Pronizius E.. (2023). Perceptions of stress and mood associated with listening to music in daily life during the COVID-19 lockdown. JAMA Netw. Open 6:e2250382. doi: 10.1001/jamanetworkopen.2022.50382, PMID: 36626171 PMC9857599

[ref8] Fletcher A. C. (1915). The study of Indian music. Proc. Natl. Acad. Sci. U. S. A. 1, 231–235. doi: 10.1073/pnas.1.4.231, PMID: 16575985 PMC1090785

[ref9] Georges P. (2017). Western classical music development: a statistical analysis of composers similarity, differentiation and evolution. Scientometrics 112, 21–53. doi: 10.1007/s11192-017-2387-x, PMID: 28725093 PMC5486899

[ref10] Gopal K. V. Champlin S. Phillips B. (2019). Assessment of safe listening intentional behavior toward personal listening devices in Young adults. Int. J. Environ. Res. Public Health 16. doi: 10.3390/ijerph16173180, PMID: 31480442 PMC6747380

[ref11] Haeyen S. Noorthoorn E. (2021). Validity of the self-expression and emotion regulation in art therapy scale (SERATS). PLoS One 16:e0248315. doi: 10.1371/journal.pone.0248315 33690731 PMC7946186

[ref12] Hahn A. Göhler A. C. Hermann C. Winkler A. (2022). Even when you know it is a placebo, you experience less sadness: first evidence from an experimental open-label placebo investigation. J. Affect. Disord. 304, 159–166. doi: 10.1016/j.jad.2022.02.043, PMID: 35181385

[ref13] Halevi-Katz D. N. Yaakobi E. Putter-Katz H. (2015). Exposure to music and noise-induced hearing loss (NIHL) among professional pop/rock/jazz musicians. Noise Health 17, 158–164. doi: 10.4103/1463-1741.155848, PMID: 25913555 PMC4918652

[ref14] Hanson D. R. Fearn R. W. (1975). Hearing acuity in young people exposed to pop music and other noise. Lancet 306, 203–205. doi: 10.1016/s0140-6736(75)90673-x 51961

[ref15] Healy K. (2016). A theory of human motivation by Abraham H. Br. J. Psychiatry 208:313. doi: 10.1192/bjp.bp.115.179622 27036694

[ref16] Kim J. Chung Y. J. (2023). A case study of group art therapy using digital media for adolescents with intellectual disabilities. Front. Psychol. 14:1172079. doi: 10.3389/fpsyt.2023.1172079, PMID: 37200905 PMC10187545

[ref17] Knoerl R. Mazzola E. Woods H. Buchbinder E. Frazier L. LaCasce A.. (2022). Exploring the feasibility of a mindfulness-music therapy intervention to improve anxiety and stress in adolescents and Young adults with Cancer. J. Pain Symptom Manag. 63, e357–e363. doi: 10.1016/j.jpainsymman.2021.11.013 34896280

[ref18] Kortesoja L. Vainikainen M. P. Hotulainen R. Merikanto I. (2023). Late-night digital media use in relation to Chronotype, sleep and tiredness on school days in adolescence. J. Youth Adolesc. 52, 419–433. doi: 10.1007/s10964-022-01703-4, PMID: 36401709 PMC9842555

[ref19] Lei H. (2022). Effect of multivoice chorus on interpersonal communication disorder. Occup. Ther. Int. 2022:6124778. doi: 10.1155/2022/6124778, PMID: 35854942 PMC9288328

[ref20] Lepping R. J. Bruce J. M. Gustafson K. M. Hu J. Martin L. E. Savage C. R.. (2019). Preferential activation for emotional Western classical music versus emotional environmental sounds in motor, interoceptive, and language brain areas. Brain Cogn. 136:103593. doi: 10.1016/j.bandc.2019.103593, PMID: 31404816 PMC6810823

[ref21] Martino S. C. Collins R. L. Elliott M. N. Strachman A. Kanouse D. E. Berry S. H. (2006). Exposure to degrading versus nondegrading music lyrics and sexual behavior among youth. Pediatrics 118, e430–e441. doi: 10.1542/peds.2006-0131, PMID: 16882784

[ref22] Mastnak W. (2016). Music in health promotion and therapeutic practice. Cultural, theoretical and clinical perspectives. Deutsche Med. Wochenschrift 141, 1845–1849. doi: 10.1055/s-0042-109418 27975358

[ref23] McFerran K. Roberts M. O'Grady L. (2010). Music therapy with bereaved teenagers: a mixed methods perspective. Death Stud. 34, 541–565. doi: 10.1080/07481181003765428, PMID: 24482859

[ref24] Neal-Barnett A. Stadulis R. Ellzey D. Jean E. Rowell T. Somerville K.. (2019). Evaluation of the effectiveness of a musical cognitive restructuring app for black Inner-City girls: survey, usage, and focus group evaluation. JMIR Mhealth Uhealth 7:e11310. doi: 10.2196/11310, PMID: 31188130 PMC6620886

[ref25] Ouergui I. Jebabli E. Delleli S. Messaoudi H. Bridge C. A. Chtourou H.. (2023). Listening to preferred and loud music enhances taekwondo physical performances in adolescent athletes. Percept. Mot. Skills 315125231178067:003151252311780. doi: 10.1177/00315125231178067, PMID: 37222224

[ref26] Paolantonio P. Cavalli S. Biasutti M. Pedrazzani C. Williamon A. (2020). Art for ages: the effects of group music making on the wellbeing of nursing home residents. Front. Psychol. 11:575161. doi: 10.3389/fpsyg.2020.575161 33329220 PMC7732664

[ref27] Perkins R. Ascenso S. Atkins L. Fancourt D. Williamon A. (2016). Making music for mental health: how group drumming mediates recovery. Psychol Well Being 6:11. doi: 10.1186/s13612-016-0048-0, PMID: 28003957 PMC5127870

[ref28] Phillips S. P. Reipas K. Zelek B. (2019). Stresses, strengths and resilience in adolescents: a qualitative study. J. Prim. Prev. 40, 631–642. doi: 10.1007/s10935-019-00570-3 31659580

[ref29] Porter S. McConnell T. McLaughlin K. Lynn F. Cardwell C. Braiden H. J.. (2017). Music therapy for children and adolescents with behavioural and emotional problems: a randomised controlled trial. J. Child Psychol. Psychiatry 58, 586–594. doi: 10.1111/jcpp.12656, PMID: 27786359

[ref30] Ren J. (2021). Pop music trend and image analysis based on big data technology. Comput. Intell. Neurosci. 2021, 4700630–4700612. doi: 10.1155/2021/4700630, PMID: 34925489 PMC8677385

[ref31] Roe D. Lysaker P. H. (2023). Meaning, recovery, and psychotherapy in light of the art of jazz. Psychiatr. Rehabil. J. doi: 10.1037/prj0000565, PMID: 36892875

[ref32] Sharma S. Sasidharan A. Marigowda V. Vijay M. Sharma S. Mukundan C. S.. (2021). Indian classical music with incremental variation in tempo and octave promotes better anxiety reduction and controlled mind wandering-a randomised controlled EEG study. Explore 17, 115–121. doi: 10.1016/j.explore.2020.02.013, PMID: 32249198

[ref33] Steele A. L. Young S. (2011). A descriptive study of Myers-Briggs personality types of professional music educators and music therapists with comparisons to undergraduate majors. J. Music. Ther. 48, 55–73. doi: 10.1093/jmt/48.1.55, PMID: 21866713

[ref34] Su P. Kong J. (2023). Implementing EMI in Chinese music classes: Students' perceived benefits and challenges. Front. Psychol. 14:1086392. doi: 10.3389/fpsyg.2023.1086392, PMID: 36998352 PMC10043248

[ref35] Taruffi L. (2021). Mind-wandering during personal music listening in everyday life: music-evoked emotions predict thought valence. Int. J. Environ. Res. Public Health 18. doi: 10.3390/ijerph182312321, PMID: 34886046 PMC8656507

[ref36] Tervaniemi M. (2023). The neuroscience of music—towards ecological validity. Trends Neurosci. 46, 355–364. doi: 10.1016/j.tins.2023.03.001, PMID: 37012175

[ref37] Vasilev M. R. Kirkby J. A. Angele B. (2018). Auditory distraction during Reading: a Bayesian Meta-analysis of a continuing controversy. Perspect. Psychol. Sci. 13, 567–597. doi: 10.1177/1745691617747398, PMID: 29958067 PMC6139986

[ref38] Wang L. Jiang N. (2022). Managing Students' creativity in music education—the mediating role of frustration tolerance and moderating role of emotion regulation. Front. Psychol. 13:843531. doi: 10.3389/fpsyg.2022.843531, PMID: 35496233 PMC9045781

[ref39] Wang X. Wei Y. Heng L. McAdams S. (2021). A cross-cultural analysis of the influence of timbre on affect perception in Western classical music and Chinese music traditions. Front. Psychol. 12:732865. doi: 10.3389/fpsyg.2021.732865, PMID: 34659045 PMC8511703

[ref40] Wang T. Zhao Y. Yin M. (2022). Analysis and research on the influence of music on students' mental health under the background of deep learning. Front. Psychol. 13:998451. doi: 10.3389/fpsyg.2022.998451, PMID: 36312155 PMC9605585

[ref41] Xia T. Sun Y. An Y. Li L. (2023). The influence of music environment on conceptual design creativity. Front. Psychol. 14:1052257. doi: 10.3389/fpsyg.2023.1052257, PMID: 36844313 PMC9946974

[ref42] Yifan Zou I. Wang W. S. (2021). Music as social bonding: a cross-cultural perspective. Behav. Brain Sci. 44:e95. doi: 10.1017/S0140525X20001326 34588046

